# Acute Fetal Hemorrhagic Shock Due to Umbilical Cord Rupture in a Term Pregnancy With Single Umbilical Artery and Velamentous Cord Insertion: A Case Report and Literature Review

**DOI:** 10.7759/cureus.69078

**Published:** 2024-09-10

**Authors:** Ayako Inatomi, Daisuke Katsura, Shinsuke Tokoro, Shunichiro Tsuji, Takashi Murakami

**Affiliations:** 1 Department of Obstetrics and Gynecology, Shiga University of Medical Science, Otsu, JPN

**Keywords:** emergency cesarean section, nuchal cord, single umbilical artery, umbilical cord rupture, velamentous cord insertion

## Abstract

Umbilical cord rupture, though rare, is a severe obstetric complication with significant implications for neonatal morbidity and mortality. We present the case of a 38-year-old primiparous female diagnosed with a single umbilical artery (SUA) and velamentous cord insertion (VCI) in late pregnancy. At 40 weeks of gestation, during labor induction, the patient suddenly experienced massive vaginal bleeding and fetal bradycardia, necessitating an emergency cesarean section. Postoperatively, it was confirmed that the umbilical cord had ruptured. The neonate required immediate and intensive resuscitation, including blood transfusion and therapeutic hypothermia. Remarkably, despite the critical initial condition, the neonate exhibited no neurological deficits and was discharged in stable condition on the 27th day.

The presence of SUA and VCI likely increased the vulnerability of the umbilical cord, predisposing it to rupture. This case emphasizes the importance of prenatal ultrasound in detecting umbilical cord abnormalities such as SUA and VCI. The early detection of these abnormalities allows for proactive management, including closer monitoring and timely surgical intervention, which are crucial for optimizing neonatal outcomes. This report provides valuable insights into the pathophysiology and management of umbilical cord rupture.

## Introduction

Umbilical cord rupture represents a relatively uncommon yet clinically significant obstetric complication, with profound implications for both maternal and neonatal outcomes [1]. This condition is characterized by the rupture of the umbilical cord either during or immediately preceding labor, leading to an abrupt cessation of fetal circulation. Such a sudden disruption in blood flow can precipitate acute fetal hypoxia, thereby markedly elevating the risk of severe neurological injury, cerebral palsy, neonatal asphyxia, and potentially neonatal death. The incidence of umbilical cord rupture is exceedingly rare, with precise prevalence rates remaining undetermined [2]. The occurrence of this complication is believed to be contingent upon a variety of factors, including the structural integrity of the umbilical cord, the mode of delivery, and fetal positioning. Primary risk factors associated with umbilical cord rupture include inherent fragility of the umbilical cord, excessive traction applied during delivery, and variations in cord length [3,4]. Clinically, umbilical cord rupture is typically identified through the sudden onset of hemorrhage or abnormalities in the fetal heart rate pattern. A precipitous decline in fetal heart rate necessitates immediate intervention, often in the form of an emergent cesarean section, as timely delivery is critical to optimizing neonatal outcomes [1]. Prognostically, the outcome of umbilical cord rupture is heavily dependent on the promptness of diagnosis and the efficacy of the subsequent intervention. The existing literature on umbilical cord rupture is sparse, with most information derived from isolated case reports. These reports often highlight a correlation between umbilical cord rupture and predisposing factors such as structural anomalies of the cord or atypical labor processes [4]. The critical importance of early detection and vigilant monitoring during labor cannot be overstated, as these are pivotal in managing this rare but potentially devastating complication.

This particular case is of notable academic interest due to the concurrent presence of a single umbilical artery (SUA) and velamentous cord insertion (VCI), both of which likely contributed to the umbilical cord rupture. The rarity and complexity of this case provide valuable insights into the pathophysiology of umbilical cord rupture, to advance the understanding of this condition within the broader obstetric and perinatal context.

## Case presentation

**Figure 1:**
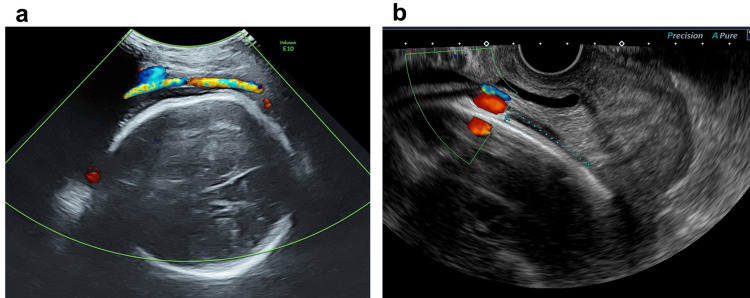
Ultrasonographic Visualization of Umbilical Cord Vessels at 40 Weeks of Gestation. (a) Transabdominal ultrasonography illustrating the course of umbilical vessels traversing the membranes along the anterior lower uterine wall. (b) Transvaginal ultrasonography demonstrating the umbilical vessels within the membranes, positioned 35 mm from the internal os on the anterior uterine wall.

**Figure 2:**
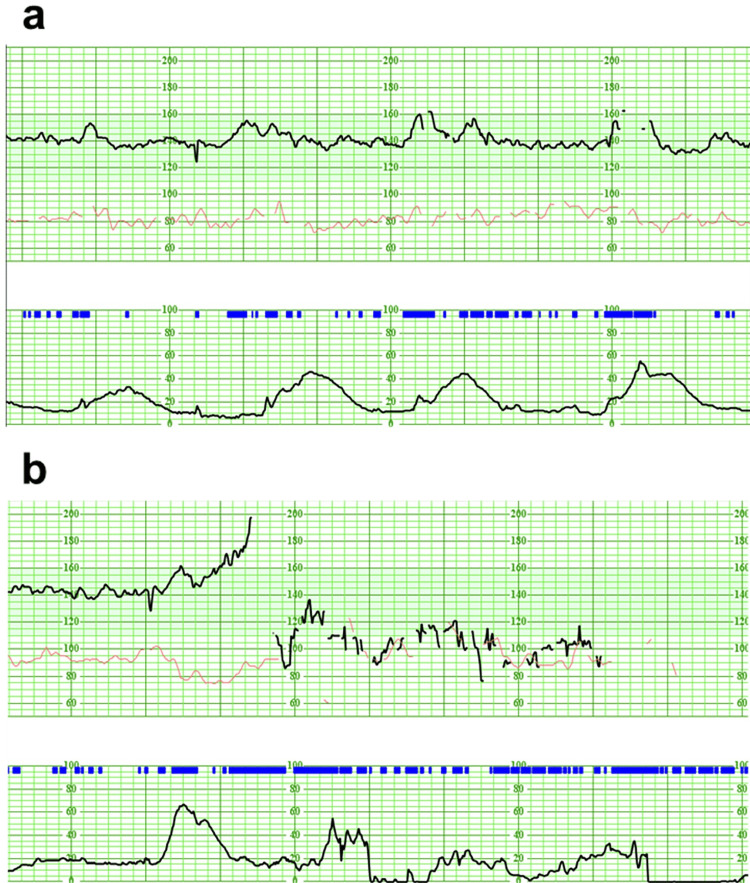
Fetal Heart Rate Monitoring During Critical Events. (a) Fetal heart rate tracings obtained during an oxytocin challenge test, showing reassuring fetal heart patterns. (b) Fetal heart rate monitoring at the onset of significant maternal vaginal bleeding, with a sudden loss of detectable fetal heart rate coinciding with the maternal hemorrhage.

The patient was a 38-year-old primiparous female with no significant medical history. She conceived spontaneously, and her pregnancy progressed without complications. She was a nonsmoker, and her prepregnancy body mass index (BMI) was 21.0 kg/m². Routine ultrasound screening in the second trimester revealed a single umbilical artery, with the insertion site of the umbilical cord into the placenta being unclear. The placenta was located on the anterior wall, with no evidence of placenta previa or low-lying placenta. Due to the presence of a single umbilical artery, she was referred to our hospital at 33 weeks of gestation. At our facility, a third-trimester ultrasound screening also failed to visualize the umbilical cord insertion site. During the 40-week antenatal checkup, a velamentous cord insertion was suspected for the first time. Color Doppler imaging demonstrated vessels traversing the membranes on the anterior lower uterine wall (Figure 1a). Transvaginal ultrasound revealed fetal vessels inserted into the membranes 35 mm from the internal os (Figure 1b). Given the absence of vasa previa, the trial of labor was planned. However, considering the risk of compression or the rupture of the membranous fetal vessels during labor, which could lead to acute fetal distress, we decided to admit the patient for an oxytocin challenge test (OCT). The OCT was performed, confirming that there was no fetal bradycardia despite the presence of painful uterine contractions (Figure 2a).On the second day of hospitalization, laminaria was used for cervical dilation due to an unfavorable cervix. On the third day, labor induction with oxytocin was initiated. As the cervix dilated to 2 cm, sudden and profuse vaginal bleeding occurred while the patient was using the restroom. Fetal heart rate was undetectable on cardiotocography (CTG) (Figure 2b), and transabdominal ultrasound revealed severe bradycardia with a fetal heart rate of approximately 30 beats per minute. An emergent cesarean section was immediately planned, and the patient was taken directly to the operating room. Under general anesthesia, a cesarean section was performed, with 18 minutes elapsing from the onset of vaginal bleeding to delivery. The maternal postoperative course was uneventful, and she was discharged on the sixth postpartum day as planned.

**Figure 3:**
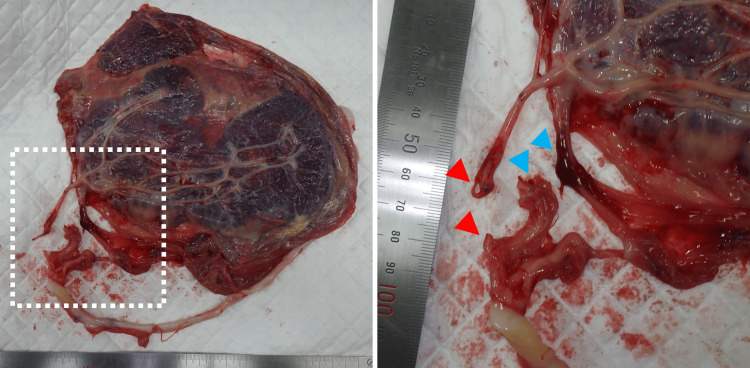
Macroscopic Examination of the Placenta. Photographic documentation of the placenta. The left panel displays a gross view, while the right panel presents an enlarged image of the region outlined by the white dotted line. The red arrow marks the rupture site of the umbilical artery, and the blue arrow indicates the rupture site of the umbilical vein.

A female infant weighing 3157 g was delivered via cesarean section. The appearance, pulse, grimace, activity, and respiration (APGAR) scores were 0, 2, and 3 at one, five, and 10 minutes, respectively. The neonate had a notably pale complexion and poor overall skin color. The umbilical cord was found wrapped around the infant's neck. There was no evidence of placental abruption, and the amniotic fluid was clear with no bloodstaining. Umbilical artery blood gas could not be obtained due to umbilical cord rupture. Blood gas analysis from capillary blood collected from the neonate's heel upon admission to the NICU revealed a pH of 6.874, hemoglobin of 7.6 g/dL (the mean level in term infants is 16.5 g/dL, with a range of ±2 SD {3.0 g/dL}), and hematocrit of 23.7% (the mean level in term infants is 51%, with a range of ±2 SD {9%}) [5]. The placenta weighed 403 g (10th percentile, 522 g, and 90th percentile, 875 g, at 41 weeks of gestation), measuring 21 × 16 × 1.5 cm [6]. The umbilical cord was 55 cm long (the mean length in term infants is 59.6 cm with a range of ±1 SD {12.6 cm}) and exhibited velamentous insertion, with the umbilical artery extending approximately 8 cm and the umbilical vein extending approximately 10 cm over the membranes without Wharton's jelly coverage [7]. The umbilical artery ruptured 3 cm from the placenta, and the umbilical vein ruptured 4 cm from the placenta (Figure 3). Histopathological examination revealed no signs of chorioamnionitis or placental abruption. The umbilical cord contained one artery and one vein, with no evidence of true knots, thrombosis, or funisitis.

**Figure 4:**
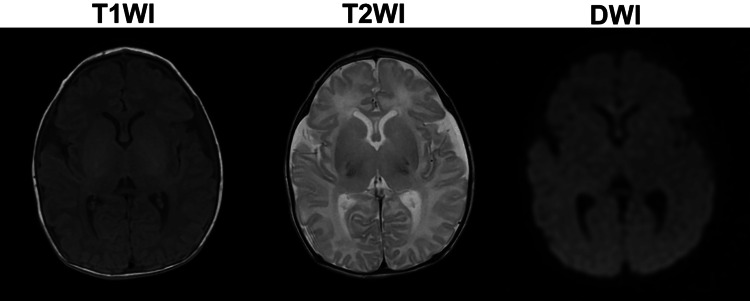
Magnetic Resonance Imaging (MRI) of the Neonatal Brain. MRI of the neonatal brain was performed on the 19th day of life and revealed no evidence of hypoxic-ischemic encephalopathy. T1WI, T1-weighted image; T2WI, T2-weighted image; DWI, diffusion-weighted image

Neonatal resuscitation was initiated immediately after birth. The neonatal heart rate was detected within five minutes of birth. The neonate received a blood transfusion and underwent therapeutic hypothermia for 72 hours. Extubation was performed on day 9 of life. Brain magnetic resonance imaging conducted on days 5 and 19 showed no abnormalities such as hypoxic-ischemic encephalopathy (Figure 4). The neonate was discharged on day 27 with no complications. Based on clinical and histological findings, it was diagnosed that the fetal vessels running over the membranes had ruptured during labor, leading to acute fetal hemorrhagic shock.

## Discussion

Umbilical cord rupture refers to a condition in which the umbilical cord ruptures due to physical forces during or immediately before labor, leading to a sudden interruption of blood flow between the fetus and the placenta. This condition can cause acute fetal hypoxia, increasing the risk of neurological injury, cerebral palsy, neonatal asphyxia, or even neonatal death [1]. Umbilical cord rupture is an extremely rare obstetric complication, and its incidence is not well documented. Umbilical cord rupture typically occurs in cases of vasa previa, where fetal vessels are located over the internal cervical os [8]. However, in this case, although there were no fetal vessels over the internal os, the most severe complication, umbilical cord rupture, occurred, prompting a review of the literature and a report of this case.

The umbilical cord is a critical structure for the exchange of oxygen and nutrients between the fetus and the mother [9]. Normally, the umbilical cord contains two umbilical arteries and one umbilical vein. The umbilical arteries carry deoxygenated blood from the fetus to the placenta, while the umbilical vein transports oxygenated blood to the fetus. The umbilical cord is typically covered by Wharton's jelly, a gelatinous substance that protects the cord's blood vessels from physical pressure and torsion. Umbilical cord rupture occurs when physical stress on the cord exceeds its tensile strength, which can result from factors such as cord vulnerability, abnormal labor progression, or excessive traction [3,4]. While the umbilical cord generally has sufficient strength, certain conditions can compromise its integrity, increasing the risk of rupture. Various abnormalities of the umbilical cord exist, including single umbilical artery (SUA), short cord, long cord, or conditions such as cord knots and abnormal cord insertion, which can heighten the vulnerability of the umbilical cord and potentially impair oxygen delivery to the fetus [10].

In this case, the presence of a single umbilical artery (SUA) likely contributed to the vulnerability of the umbilical cord. SUA occurs in approximately 0.5%-5% of all pregnancies and can result from a congenital failure to form the second umbilical artery during embryonic vasculogenesis or from secondary occlusion or atrophy [11]. Factors such as maternal smoking, diabetes, or drug use can increase the incidence of SUA, but none were present in this case [12]. SUA is associated with chromosomal abnormalities and other congenital malformations, particularly trisomies 18 and 21, and fetuses with SUA are at higher risk of growth restriction due to compromised blood flow [11]. Additionally, SUA is often linked to congenital anomalies of the heart, kidneys, and gastrointestinal tract, making detailed ultrasound evaluations essential [13]. Moreover, in cases of SUA, there is often a reduction in Wharton's jelly, which normally provides cushioning and protection to the vessels within the umbilical cord [14]. A decreased amount of Wharton's jelly can leave the umbilical vessels more exposed and susceptible to damage, potentially increasing the risk of rupture. Although this case did not present fetal growth restriction or other malformations, the reduced protection provided by Wharton's jelly in the presence of SUA may have contributed to the increased risk of cord rupture observed in this instance.

Moreover, velamentous cord insertion (VCI) in this case may have also played a role in the cord's vulnerability. When the umbilical cord does not attach normally to the placenta but instead inserts into the membranes, this abnormal attachment, such as marginal or velamentous insertion, results in some of the cord's vessels being unprotected by Wharton's jelly, making them more susceptible to rupture [3]. The incidence of velamentous insertion is approximately 1%-2%, with a higher prevalence in multiple pregnancies. VCI can compromise placental blood flow, increasing the risk of fetal growth restriction [4]. The incidence of preterm birth and intrauterine fetal demise is reported to be higher in VCI cases compared to normal cord insertion cases, likely due to the ease with which these vessels can be compressed or ruptured during uterine contractions or the rupture of membranes. When the cord vessels overlap the internal os, it is termed vasa previa, which carries a significant risk of fatal hemorrhage during delivery, typically warranting planned cesarean delivery [15]. Although umbilical cord rupture is usually associated with vasa previa, this case demonstrates that umbilical cord rupture can occur even without vasa previa. Additionally, while umbilical cord rupture is often reported to occur during the rupture of membranes, this case occurred without the rupture of membranes. One other case of cord rupture associated with velamentous cord insertion has been reported, where vasa previa was absent, and the membranes were intact [1]. This case highlights the potential for umbilical cord rupture in velamentous insertions, even in the absence of vasa previa.

Furthermore, in our case, the umbilical cord was wrapped tightly around the fetal neck once, potentially contributing to excessive traction on the cord and subsequent rupture. The nuchal cord, where the cord wraps around the fetus's neck or other body parts, occurs in approximately 10%-29% of all pregnancies, with the risk increasing as the number of cord loops increases [16]. A nuchal cord can compromise oxygen delivery to the fetus due to cord compression [17]. Additionally, a tightly wrapped nuchal cord, as observed in this case, can lead to excessive stretching or compression of the cord, increasing the risk of vascular injury and, in severe cases, cord rupture.

The evaluation of cord vulnerability is often performed via ultrasound during pregnancy. The mid-trimester (18-22 weeks) is the most appropriate time for screening umbilical cord abnormalities, as the cord's structure is sufficiently developed for assessment. Ultrasound can easily confirm the number of umbilical arteries and the location of cord insertion. Color Doppler ultrasound is useful for visualizing blood flow within the umbilical cord, confirming the absence of one of the typically present two umbilical arteries [18,19]. SUA diagnosis is confirmed by observing the absence of one artery in the transverse section of the cord and the presence of a single artery emerging from the fetal bladder. If velamentous insertion is identified, additional evaluation using color Doppler in transvaginal ultrasound is recommended to assess for vasa previa. A systematic review of VCI screening indicated that while third-trimester transabdominal ultrasound has high specificity and positive predictive value, its sensitivity is only 62.5% [3]. In this case, the velamentous insertion was missed in both mid- and late-pregnancy screenings, highlighting the need for a more careful observation of the cord insertion site. In particular, based on our experience from this case, it is considered essential to always assess the cord insertion site in cases with SUA. Despite the late detection at 40 weeks, identifying the abnormal cord insertion before delivery significantly contributed to the favorable neonatal outcome.

Umbilical cord rupture is often detected based on clinical symptoms such as sudden bleeding, abnormal fetal heart rate (particularly sudden bradycardia), and decreased or absent fetal movement, which result from the acute interruption of fetal oxygen supply [1]. The bleeding typically originates from the fetal side rather than the maternal side, leading to neonatal anemia or shock. When umbilical cord rupture is suspected, an emergency cesarean section must be performed immediately. Rapid delivery via cesarean section is the only means of minimizing fetal hypoxia. Postdelivery, the neonate may require aggressive management, including resuscitation and blood transfusion, depending on the clinical condition. While a sinusoidal pattern on CTG is a hallmark of fetal anemia, the fetal response to sudden massive hemorrhage is often persistent bradycardia. A delay of more than 20 minutes from the onset of fetal bradycardia to delivery is significantly associated with fetal acidosis (pH < 7) [20]. Early detection and prompt cesarean section increase the likelihood of neonatal survival, but delayed intervention raises the risk of severe neurological sequelae or death. In this case, the pre-delivery knowledge of SUA and abnormal cord insertion allowed for the early diagnosis of cord rupture and a rapid response, with delivery occurring 18 minutes after the onset of vaginal bleeding.

## Conclusions

Umbilical cord rupture is an extremely rare obstetric complication that can cause sudden fetal hypoxia, significantly increasing the risk of severe neurological damage or neonatal death. In this case, the presence of a single umbilical artery, velamentous cord insertion, and nuchal cord may have contributed to the cord rupture. In similar situations where these risk factors are present, attempting vaginal delivery may not be advisable. These abnormalities can potentially be detected early through ultrasound examination during pregnancy, highlighting the importance of evaluating cord insertion and the umbilical arteries in the mid-trimester. Rapid response to the information obtained about the umbilical cord significantly influences neonatal survival and prognosis.
